# Genomic selection of purebred animals for crossbred performance in the presence of dominant gene action

**DOI:** 10.1186/1297-9686-45-11

**Published:** 2013-04-26

**Authors:** Jian Zeng, Ali Toosi, Rohan L Fernando, Jack CM Dekkers, Dorian J Garrick

**Affiliations:** 1 Department of Animal Science and Center for Integrated Animal Genomics, Iowa State University, Ames, IA, USA; 2, Monsanto Company, Ankeny, IA, USA

## Abstract

**Background:**

Genomic selection is an appealing method to select purebreds for crossbred performance. In the case of crossbred records, single nucleotide polymorphism (SNP) effects can be estimated using an additive model or a breed-specific allele model. In most studies, additive gene action is assumed. However, dominance is the likely genetic basis of heterosis. Advantages of incorporating dominance in genomic selection were investigated in a two-way crossbreeding program for a trait with different magnitudes of dominance. Training was carried out only once in the simulation.

**Results:**

When the dominance variance and heterosis were large and overdominance was present, a dominance model including both additive and dominance SNP effects gave substantially greater cumulative response to selection than the additive model. Extra response was the result of an increase in heterosis but at a cost of reduced purebred performance. When the dominance variance and heterosis were realistic but with overdominance, the advantage of the dominance model decreased but was still significant. When overdominance was absent, the dominance model was slightly favored over the additive model, but the difference in response between the models increased as the number of quantitative trait loci increased. This reveals the importance of exploiting dominance even in the absence of overdominance. When there was no dominance, response to selection for the dominance model was as high as for the additive model, indicating robustness of the dominance model. The breed-specific allele model was inferior to the dominance model in all cases and to the additive model except when the dominance variance and heterosis were large and with overdominance. However, the advantage of the dominance model over the breed-specific allele model may decrease as differences in linkage disequilibrium between the breeds increase. Retraining is expected to reduce the advantage of the dominance model over the alternatives, because in general, the advantage becomes important only after five or six generations post-training.

**Conclusion:**

Under dominance and without retraining, genomic selection based on the dominance model is superior to the additive model and the breed-specific allele model to maximize crossbred performance through purebred selection.

## Background

Numerous studies have shown encouraging results of applying genomic selection (GS) in purebred populations [1-6]. However, except for dairy cattle, most animals used in livestock production systems are crossbreds, with advantages of heterosis and breed complementarity. For such systems, the breeding goal in purebreds should be to optimize the performance of crossbred descendents.

GS has advantages in selection for crossbred performance over conventional methods [7-9] as reported in [9]. In particular, training on crossbred data for GS accounts for genetic differences between purebred and crossbred animals and genotype by environment effects. Different GS models have been proposed and used to select purebreds for crossbred performance [8-11]. In most studies, additive gene action or perfect knowledge of gene substitution effects or both have been assumed. It has been argued that dominance is the likely genetic basis of heterosis [12,13], therefore explicitly including dominance in the GS model may be beneficial for selection of purebreds for crossbred performance.

Allele substitution effects are a function of additive and dominance effects, and of allele frequencies [12]. In the case of crossbred records, Dekkers [8] proposed a model that fits breed-specific allele substitution effects (BSAM), where the substitution effects at the QTL (quantitative trait loci) for paternal and maternal alleles would be different if the parental breeds differ in allele frequencies at the QTL. It has been shown that, when additive and dominance effects of the QTL are known without error, BSAM can give greater response to selection than the usual additive model that fits a common substitution effect for each QTL [10,11]. When the QTL genotype is not observed and marker genotypes are fitted in the model, linkage disequilibrium (LD) between SNPs and the QTL may not be consistent between the parental breeds. This difference in LD will also contribute to differences in allele substitution effects between breeds. When SNP effects must be estimated, the advantage of fitting BSAM over the additive GS model was not always observed under additive inheritance [9], which suggests that differences in LD between breeds may not be as important as the presence of dominant gene action in practice.

Dekkers [14] showed that, to maximize performance in the first generation of crossbreds, the allele substitution effect for an identified QTL must be derived based on allele frequencies in the selected mates. However, allele frequencies of selected mates cannot be observed prior to computation of the substitution effects that are needed for selection. Thus, Dekkers [14] proposed an iterative algorithm to compute substitution effects based on selected mates.

A model that explicitly includes dominance effects (the dominance model) provides estimates of both additive and dominance effects and therefore enables the computation of allele substitution effects using appropriate allele frequencies. Once estimates of SNP effects are obtained from training, they can be successively applied over generations with updated allele frequencies to develop prediction equations specific to that generation.

The BSAM model gives allele substitution effects for each parental breed that depend on allele frequencies among individuals from the other breed that were used to produce the training population. It is not straightforward to apply the iterative algorithm of Dekkers to BSAM. In addition, the substitution effects from BSAM cannot be used to recompute the appropriate substitution effects in the subsequent generations, as allele frequencies change due to selection. Furthermore, breed origin of SNP alleles must be known or inferred for the BSAM model [8,10], but such knowledge is not needed for the dominance model.

The primary objective of this study was to assess the performance of the dominance model in comparison with the additive model or BSAM for GS on purebreds for crossbred performance. Substitution effects from the dominance model were computed based on allele frequencies of unselected mates. Ibáne~z-Escriche et al. [9] compared BSAM to the additive model under additive gene action alone and Kinghorn et al. [10,11] made this comparison with dominant gene action when QTL effects were assumed known. Thus, a secondary objective of this study was to compare BSAM to the additive model under dominance when SNP effects must be estimated. Model performance was evaluated by computer simulation based on response to 20 generations of selection in a two-way crossbreeding program.

## Methods

### Simulations

Comparisons between the dominance, BSAM and additive models were made for four scenarios of gene action. To clearly detect an advantage of including dominance in the model, the dominance variance *V*_*D*_ and heterosis *H* were chosen to be large in scenario 1, allowing for overdominance. In scenarios 2 and 3, *V*_*D*_ and *H* were restricted to more realistic values with (scenario 2) or without (scenario 3) overdominance. In scenario 4, *V*_*D*_ was reduced to zero to examine any disadvantage of using the dominance model when gene action is purely additive. Changes to *V*_*D*_ and *H* were achieved by changing the size and proportion of beneficial dominance effects. Other parameters, including locus positions and LD between loci and allele frequencies were held constant between the four scenarios. A total of 16 random simulations were carried out for each scenario.

#### Genome and trait phenotypes

A genome was simulated with either one or ten chromosomes. Each chromosome was one Morgan and consisted of 100 randomly distributed QTL and 1000 SNP markers that were almost evenly spaced. All loci were biallelic with starting allele frequencies of 0.5 and a reversible mutation rate of 2.5×10^-5^. A binomial map function was used to model recombination with interference on a chromosome [15]. A base population of 500 unrelated individuals was randomly mated for 1000 discrete generations to create LD between loci in the founders of different breeds.

The additive effect *a* of a QTL is defined as half the difference in genotypic value between alternate homozygotes and the dominance effect *d* as the deviation of the value of the heterozygote from the mean of the two homozygotes [12]. Bennewitz and Meuwissen [16] evaluated QTL mapping results from many studies in pigs for meat quality and carcass traits and concluded that an exponential distribution with rate parameter 5.81 is an adequate “generating mechanism” for the absolute values of the additive effects of QTL. That distribution was used here to generate the unsigned value of the additive effect for each QTL, and a positive or negative sign was assigned to each additive effect with equal probability. Although the relationship between additive and dominance effects of QTL has been studied [16-18], a consistent relationship has not been observed. Thus, in scenarios 1 and 2 with overdominance, we assumed, for simplicity, that the dominance effects were independent of additive effects. The absolute values of dominance effects were independently sampled from the same exponential distribution as was used for the additive effects. This was considered reasonable because the exponential distribution has a high probability for the occurrence of small effects, which is also plausible for dominance effects. In scenario 3 with no overdominance, the dominance effect of a QTL was sampled from a uniform distribution that ranged from zero to the absolute value of the QTL’s additive effect. In order for the trait to manifest positive heterosis, only 30% of the sampled dominance effects were negative in scenarios 1 and 2, and this fraction was further reduced to 20% in scenario 3 for the sake of no overdominance. The resulting distribution of dominance coefficients, defined as the ratio of the dominance effect over the absolute value of additive effect, was similar to what has been observed for real data [16].

The QTL effects were scaled (as described in AppendixAppendix A) such that the relative contributions of the additive and dominance effects to the genetic variability of the trait were 2:1, 4:1 and 1:0 in the scenarios where *V*_*D*_ was set to be large, realistic or null, respectively. After scaling, 40-45% of QTL showed partial dominance and 30-35% overdominance when overdominance was present. Trait phenotypes were simulated by adding a standard normal residual effect to the genotypic value of each animal. The variance of the residual effects was chosen such that broad sense heritability $h_{\text{bs}}^{2}$ of the trait was 0.5 in the founders. As a result, narrow sense heritability $h_{\text{ns}}^{2}$ was 0.33 for the scenario with large *V*_*D*_, 0.4 with realistic *V*_*D*_, and 0.5 with *V*_*D*_ = 0.

#### Breed formation

**Figure 1:**
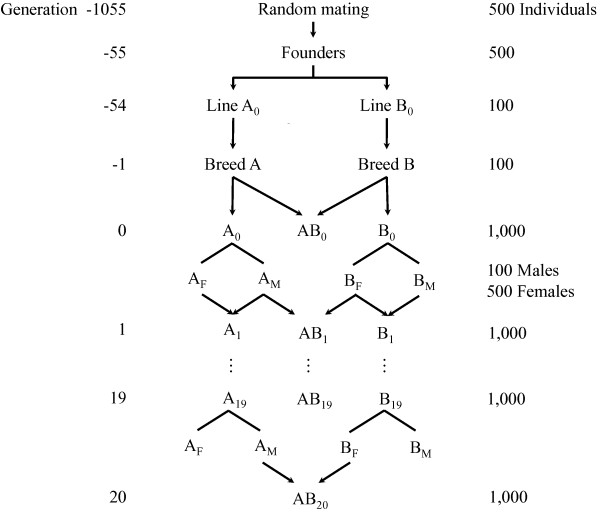
**Schematic representation of the simulated population history and the two-way crossbreeding program.** The crossbreeding program consisted of 20 generations of purebred selection for crossbred performance; crossbred AB_0_ is the training population; A_*M*_ and B_*M*_ represent the selected breed A and B males, A_*F*_ and B_*F*_ the selected breed A and B females; lines with arrows denote reproduction, while lines without arrows denote selection.

Breeds A and B were simulated by randomly sampling 100 animals from the founders in generation -55 and random mating for 54 additional generations to mimic recent breed formation (Figure 1). In the founder generation, 100 QTL and 1000 SNPs were randomly chosen from those with a minor allele frequency greater than 0.1. To guarantee that the number of such loci to choose from would be sufficient, five times as many loci were simulated in the base population. This procedure ensured that most QTL chosen to define the trait and SNPs chosen for inclusion in the analysis segregated in both breeds.

In generation -1, the genetic disparity between breeds was due to differences in LD and in allele frequencies. Averaged over simulations, the heterozygosity of each breed was about 0.3, and the mean difference in allele frequencies between breeds was about 0.3. In each simulation, while the same set of QTL characterized the trait, the contribution of QTL effects to the phenotypic variability differed between breeds due to disparities in allele frequencies. Between simulations, the observed values of variance components in a given breed varied due to genetic drift during the 54 generations of random mating after breed separation.

In livestock, dominance can explain up to about 10% of phenotypic variation [19] and heterosis from a breed cross can be up to 10% [20]. In scenario 1, where *V*_*D*_ was large, *V*_*D*_ was on average 16.7% in the pure breeds and *H* was on average 31%. Under more realistic settings, ${\bar{V}}_{D}=10$% and $\bar{H}=9.1$% in scenario 2, where overdominance was allowed, and ${\bar{V}}_{D}=4.5$% and $\bar{H}=8.4$% in scenario 3, where overdominance was not allowed. When the genome was extended from one to ten chromosomes, these values were kept about the same by reducing the proportion of beneficial dominance effects.

#### Crossbreeding program

A two-way crossbreeding program with 20 generations of selection was simulated, as illustrated in Figure 1. The goal was to improve crossbred performance through selection in both parental breeds, starting from 1000 animals of sire breed (A) and 1000 animals of dam breed (B) in generation 0. The selection criteria was the rank of the individual’s genomic estimated breeding values (GEBV). The SNP effects for the prediction of GEBV were estimated only once, using 1000 crossbred AB_0_ animals in generation 0. These estimates of SNP effects were then repeatedly applied to predict GEBV in the following 20 generations of selection. In generation 1 through 20, 600 animals were selected from 1000 candidates in each parental breed based on their GEBV, of which the top 100 were used as males and the other 500 as females. As described in detail later, with the dominance model, GEBV were based on the breed-specific allele substitution effects that were recomputed for each generation, using allele frequencies in the opposite breed in that generation. The selected animals were randomly mated within each breed to produce 1000 purebred replacement animals for the next generation. Meanwhile, the 100 selected males of breed A were also randomly mated to the 500 selected females of breed B to produce 1000 crossbred progeny. The phenotypic mean of crossbreds was computed in each generation of selection (AB_1_ – AB_20_) to evaluate the cumulative response to selection.

Starting from the same set of purebred selection candidates in generation 0, the subsequent 20 generations of selection were repeated 100 times to increase power to detect differences between the GS models in cumulative response. A mixed linear model (see Appendix B) was used to test for differences between GS models by generation 20. Note that the 16 random simulations each with 100 repetitions account for differences in purebreds due to genetic drift. In sum, the collected data consisted of 1600 observations in each generation of selection.

The cumulative response in the *i*^*th*^ generation of selection, where *i* = 1, …, 20, was computed as

$$R_{i}=\frac{\mu_{i}-\mu_{0}}{\sigma_{0}},$$

where *μ*_*i*_ is the phenotypic mean of crossbreds in generation *i* and *σ*_0_ is the phenotypic standard deviation of crossbreds in generation 0.

### Statistical models

#### Additive model

The following mixed linear model was used to estimate SNP effects assuming additive gene action:

$$y_{i}=\mu +\overset{k}{\underset{j=1}{\sum }}X_{\text{ij}}\alpha_{j}+e_{i},$$

where *y*_*i*_ is the phenotype of animal *i*, *μ* is the overall mean, *X*_*ij*_ is the copy number of a given allele of SNP *j* centered by the mean, *α*_*j*_ is the allele substitution effect for SNP *j*, and *e*_*i*_ is the residual effect for animal *i*. The prior specification for model parameters and the sampling strategy followed the BayesC *Π* method proposed by [6]. In order to concentrate the signal and reduce noise, only a proportion of SNPs was assumed to have a non-null effect. Conditional on $\sigma_{\alpha }^{2}$, the variance of random substitution effects for all SNPs, *α*_*j*_ had a mixture prior of a normal distribution and a point mass at zero:

(1) $$\alpha_{j}|\sigma_{\alpha }^{2}=\left\{\begin{array}{ll} 0 & \text{with probability}\;\Pi \\ ∼N(0,\sigma_{\alpha }^{2}) & \text{with probability}\;1-\mathrm{\Pi .} \end{array}\right)$$

The proportion *Π* of SNPs that have null effects on the trait was considered unknown, with a uniform prior between 0 and 1. A scaled inverse Chi-square distribution with degrees of freedom *ν*_*α*_ = 4 and scale parameter $S_{\alpha }^{2}$ was specified as a prior for $\sigma_{\alpha }^{2}∼\nu_{\alpha }S_{\alpha }^{2}\chi_{\nu_{\alpha }}^{-2}$. The value of $S_{\alpha }^{2}$ was chosen based on the following relationship between the expectation of a scaled inverse Chi-square variable and its scale parameter:

(2) $$E(\sigma^{2})=\frac{S^{2}\nu }{\nu -2},$$

and $E(\sigma_{\alpha }^{2})$ was obtained following [21] such that $E(\sigma_{\alpha }^{2})=\frac{V_{A}}{k(1-\Pi_{0})E(2\text{pq})}$, where *k* is the total number of SNPs, *Π*_0_ is the chosen probability that a SNP has no effect prior to the analysis, *p* = 1 - *q* is the allele frequency, and *V*_*A*_ is the additive genetic variance for the trait that is explained by all SNPs. The residual *e*_*i*_ had a normal prior with variance also following a scaled inverse Chi-square distribution: $e_{i}|\sigma_{e}^{2}∼N(0,\sigma_{e}^{2})$ and $\sigma_{e}^{2}∼\nu_{e}S_{e}^{2}\chi_{\nu_{e}}^{-2}$, where *ν*_*e*_ = 4. The value of $S_{e}^{2}$ was obtained from (2), with $E(\sigma_{e}^{2})=V_{E}$, where *V*_*E*_ is the residual variance that cannot be explained by the SNPs. True values were given to *V*_*A*_ and *V*_*E*_ in this study.

#### Dominance model

The dominance model, as shown below, simultaneously fits additive and dominance effects of SNPs:

(3) $$y_{i}=\mu +\overset{k}{\underset{j=1}{\sum }}(X_{\text{ij}}a_{j}+W_{\text{ij}}d_{j})+e_{i},$$

where *y*_*i*_, *μ*, *X*_*ij*_ are as defined in the additive model, *W*_*ij*_ is the indicator variable for the heterozygous genotype of SNP *j* that is centered by its mean, *a*_*j*_ is the additive effect, and *d*_*j*_ the dominance effect for SNP *j*, and *e*_*j*_ is the residual. Given the assumption that epistasis is absent, the residual term in the dominance model only contains non-genetic effects, while that of the additive model also includes dominance deviations. The model specification for the dominance model is similar to that of the additive model. Conditional on *Π*_*a*_ (the probability that *a*_*j*_ is zero) and $\sigma_{a}^{2}$ (the variance of *a*_*j*_ when it is nonzero), the prior for *a*_*j*_ is a mixture of normals, as given in the additive model (1). Similarly, the prior for *d*_*j*_ is also a mixture of normals, given *Π*_*d*_ and $\sigma_{d}^{2}$, with corresponding definitions. However, in order to account for the directionality of dominance, the normal component of the prior for *d*_*j*_ has an unknown nonzero mean *μ*_*d*_:

(4) $$d_{j}|\mu_{d},\sigma_{d}^{2}=\left\{\begin{array}{ll} 0 & \text{with probability}\;\Pi_{d}\\ ∼N(\mu_{d},\sigma_{d}^{2}) & \text{with probability}\;1-\Pi_{d}. \end{array}\right)$$

For convenience, the prior of *μ*_*d*_ was assumed to depend on the variance:

$$\mu_{d}|\sigma_{d}^{2}∼N(\eta ,\sigma_{d}^{2}/\phi ),$$

where *η* is our prior belief about *μ*_*d*_ and *ϕ* is the “prior sample size”, which expresses the strength of the prior belief in terms of $\sigma_{d}^{2}$. Here, *ϕ* was set to 10, a small number relative to 100 QTL, to allow the data to “dominate” the posterior distribution of *μ*_*d*_. The value of *η* was chosen as described below.

Let the allele frequency at SNP *j* be $p_{j}^{S}$ in sires and $p_{j}^{D}$ in dams in generation -1. Assuming Hardy-Weinberg equilibrium in the parental populations, heterosis (*H*) in the training crossbred AB_0_ is a function of the dominance effects and the difference in allele frequencies in the parental populations ($\Delta =p_{j}^{S}-p_{j}^{D}$) [12]:

$$H=\underset{j}{\sum }d_{j}\Delta^{2}.$$

Assuming independence between dominance effects and allele frequencies and ignoring selection, this can be written as

$$H=k(1-\Pi_{d,0})E(d)E(\Delta^{2}),$$

where *Π*_*d*,0_ is a chosen value for the proportion of SNPs that have nonzero dominance effects. Assuming that each QTL is associated with at least one SNP, *Π*_*d*,0_ should be at most 0.9, as 100 QTL and 1000 SNPs were simulated. Rearranging gives

$$\eta =E(d)=\frac{H}{k(1-\Pi_{d,0})E(\Delta^{2})}.$$

The variance components $\sigma_{a}^{2}$ and $\sigma_{d}^{2}$ were assumed to have independent scaled inverse Chi-square distributions. As shown in (2), specification of the hyper parameters $S_{a}^{2}$ and $S_{d}^{2}$ requires knowing $E(\sigma_{a}^{2})$ and $E(\sigma_{d}^{2})$. The following describes how $E(\sigma_{a}^{2})$ or $E(\sigma_{d}^{2})$ were computed based on the known quantities *V*_*A*_ and *V*_*D*_.

Given independence between SNPs holds and in the absence of selection [12]:

(5) $$V_{D}=\sum_{j}{\left(2p_{j}q_{j}d_{j}\right)}^{2}.$$

Assuming independence between effects and allele frequencies, this can be written as

$$\begin{array}{lll} V_{D} & =k(1-\Pi_{d,0})E[{\left(2\text{pq}\right)}^{2}]E(d^{2})\; & \;\\ & =k(1-\Pi_{d,0})E[{\left(2\text{pq}\right)}^{2}]\{(1+1/\phi )\sigma_{d}^{2}+{\left[E(d)\right]}^{2}\}.\; & \; \end{array}$$

Rearranging and replacing E(*d*) by *η* gives

(6) $$\sigma_{d}^{2}=(\frac{V_{D}}{k(1-\Pi_{d,0})E[{\left(2\text{pq}\right)}^{2}]}-\eta^{2})/(1+1/\phi ).$$

Also [12]:

(7) $$V_{A}=\underset{j}{\sum }(2p_{j}q_{j}\alpha_{j}^{2}).$$

Under the same assumptions as made in (5), in addition to additive effects having mean zero and being independent of dominance effects, this becomes

(8) $$V_{A}=k(1-\Pi_{a,0})E(2\text{pq})E(\alpha^{2}),$$

where

$$\begin{array}{lll} E(\alpha^{2}) & =E\{{\left[a+(1-2p)d\right]}^{2}\}\; & \;\\ & =\sigma_{a}^{2}+E[{\left(q-p\right)}^{2}](\sigma_{d}^{2}+\eta^{2}).\; & \; \end{array}$$

Substituting this in (8) and rearranging gives

(9) $$\sigma_{a}^{2}=\frac{V_{A}}{k(1-\Pi_{a,0})E(2\text{pq})}-E[{\left(1-2p\right)}^{2}](\sigma_{d}^{2}+\eta^{2}).$$

Values for $S_{a}^{2}$ and $S_{d}^{2}$ can now be calculated by substituting (9) and (6) in (2), respectively.

#### Breed-specific SNP allele model

As shown in [9] (with a slightly different notation), BSAM fits SNP allele states in the following model:

(10) $$y_{i}=\mu +\overset{k}{\underset{j=1}{\sum }}(X_{\text{ij}}^{A}\alpha_{j}^{A}+X_{\text{ij}}^{B}\alpha_{j}^{B})+e_{i},$$

where $X_{\text{ij}}^{A}$ and $X_{\text{ij}}^{B}$, with value (0, 1), are the breed-specific copy numbers of a given allele at SNP *j* of breed origin *A* or *B* that animal *i* received from its sire or dam, and $\alpha_{j}^{A}$ and $\alpha_{j}^{B}$ are the breed-specific substitution effects for the alleles of breed origin *A* and *B*. The other parameters are defined as in the additive and dominance models. In BSAM, the SNP allele effects have breed-specific variances $\frac{}{}\sigma_{\alpha^{A}}^{2}$ and $\frac{}{}\sigma_{\alpha^{B}}^{2}$, and breed-specific parameters $\frac{}{}\Pi_{\alpha^{A}}$ and $\frac{}{}\Pi_{\alpha^{B}}$. The same prior as used in the additive model is used for $\frac{}{}\sigma_{\alpha^{A}}^{2}$ and $\frac{}{}\sigma_{\alpha^{B}}^{2}$.

### Inference for model parameters

Markov chain Monte Carlo (MCMC) sampling was used to draw inferences from the posterior distributions of parameters. Gibbs sampling was used to sample parameters from their full conditional distributions, which are derived for some key model parameters in Appendix C. Since the implementation of a Gibbs sampler in the additive model has been well described by [6], here we focus on the algorithm for the dominance model. The decision to include a SNP in the model was separately sampled for the additive and dominance effects in the dominance model. Similarly, in BSAM, the decision to include a SNP in the model was separately sampled for the sire and dam breed-specific allele substitution effects.

The analyses were implemented by modifying *GenSel*[22] to allow dominance and allele specific effects. The Markov chain used for inference consisted of 11 000 samples, with the first 1000 discarded as a burn-in. Longer chains did not improve prediction accuracy. Parameters were estimated from the mean of the resulting 10 000 posterior samples.

### True and genomic estimated breeding values

For animal *i* from breed *r*, the true breeding value is given by

(11) $$\text{TB}V_{i}^{r}=\overset{m}{\underset{t=1}{\sum }}T_{\text{it}}\alpha_{t}^{r},$$

where *T*_*it*_ is the QTL genotype, coded as 0, 1, or 2, and $\alpha_{t}^{r}$ is the true allele substitution effect for QTL *t*, and the GEBV is given by

(12) $$\text{GEB}V_{i}^{r}=\overset{k}{\underset{j=1}{\sum }}Z_{\text{ij}}{\hat{\alpha }}_{j}^{r},$$

where *Z*_*ij*_ is the marker genotype and ${\hat{\alpha }}_{j}^{r}$ is the estimated allele substitution effect for SNP *j*. The definition of $\alpha_{t}^{r}$ for a purebred animal with a breeding goal of maximizing the performance of the crossbred descendents is described next.

Suppose *Q*_1_ and *Q*_2_ are two alleles at a QTL. Let *p*^*S*^ denote the frequencies of *Q*_2_ in the sire breed and *p*^*D*^ denote the frequencies of *Q*_2_ in the dam breed. The genotypic values (G) of genotypes *Q*_1_*Q*_1_,*Q*_1_*Q*_2_ and *Q*_2_*Q*_2_ are 0,*a* + *d* and 2*a*, respectively. The average effect of a *Q*_1_ allele from the sire is defined as the expected genotypic value of a crossbred offspring that received *Q*_1_ from the sire minus the crossbred population mean. Let *S* denote the allele that animal *i* inherited from its sire. Based on the above definition,

$$\begin{array}{lll} \;\alpha_{1}^{S} & =E(G|S=Q_{1})-\mu \; & \;\\ & =(a+d)p^{D}-\mu .\; & \; \end{array}$$

Similarly, the average effect of a *Q*_2_ allele from the sire is $\alpha_{2}^{S}=(a+d)(1-p^{D})+2ap^{D}-\mu$. The difference between the two average effects gives the substitution effect for the sire:

$$\begin{array}{lll} \;\alpha^{S} & =\alpha_{2}^{S}-\alpha_{1}^{S}\; & \;\\ & =a+(1-2p^{D})d.\; & \; \end{array}$$

Similarly, the substitution effect for the dam is *α*^*D*^ = *a* + (1 - 2*p*^*S*^)*d*. As a result, the allele substitution effects for a purebred parent used for crossbreeding are breed-specific and defined in terms of the allele frequencies in the breed of the other parent.

In summary, for a purebred *r*, $\alpha_{t}^{r}$ in (11) is defined as

(13) $$\alpha_{t}^{r}=a_{t}+(1-2p_{t}^{r^{\prime }})d_{t},$$

where *r*’ is the breed of the other parent of the crossbreds. In BSAM, $\alpha_{j}^{r}$ is directly estimated for prediction of $\text{GEB}V_{i}^{r}$ in (12), while it is indirectly estimated from the dominance model by combining the estimates of *a*_*j*_ and *d*_*j*_ with the current value of $p_{j}^{r^{\prime }}$ from breed *r*’ in (13). The additive model does not estimate breed-specific substitution effects. Instead, it estimates a common *α*_*j*_ for SNP *j*, which uses the allele frequency in the crossbreds used for training.

## Results

### Cumulative response to selection

**Figure 2:**
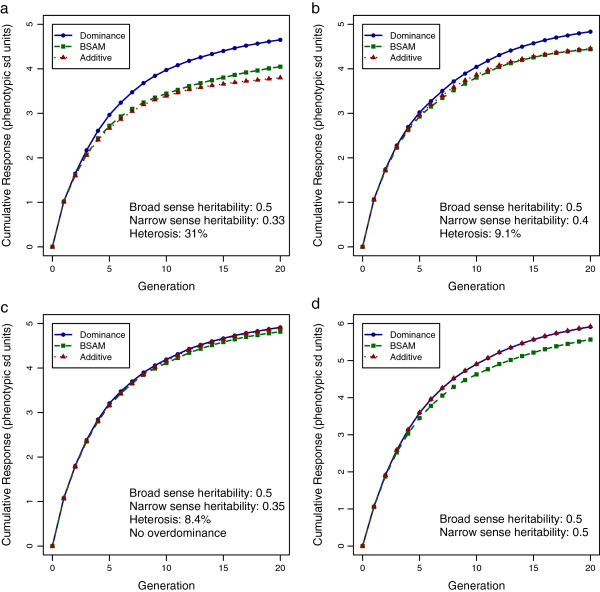
**Cumulative response to genomic selection with one chromosome.** Cumulative response to GS was computed using the dominance model, BSAM and the additive model in the four scenarios, when there was one chromosome, 100 QTL and 1000 SNPs; the plotted cumulative responses are means from 1600 replicates, standardized by the phenotypic standard deviation of crossbreds in generation 0: (**a**) results from scenario 1, where *V*_*D*_ was large with overdominance, (**b**) results from scenario 2, where *V*_*D*_ was realistic with overdominance, (**c**) results from scenario 3, where *V*_*D*_ was realistic without overdominance and (**d**) results from scenario 4, where dominance was absent.

Figure 2 depicts cumulative responses to GS for the additive, BSAM and dominance models under the four scenarios, when the genome consisted of one chromosome, 100 QTL and 1000 SNPs. The cumulative response to selection at generation 20 by the BSAM and the dominance model compared to the additive model is also given in Table 1. In scenario 1, where the dominance model was most favored, the dominance model had a substantially greater cumulative response than BSAM and the additive model. By generation 20, the dominance model had additional responses of 14.9% over BSAM and of 22.4% over the additive model (*P* < 10^-16^). In scenario 2, however, this advantage was reduced, since the proportion of dominance variance and heterosis decreased from 16.7% and 31% to about 10% for both. As a result, in generation 20, the advantage of the dominance model was reduced from 14.9% to 8.9% for BSAM and from 22.4% to 8.6% for the additive model. However, the differences in the cumulative response at generation 20 between the dominance model and the BSAM and additive model were still significant (*P* < 10^-9^). The advantage of BSAM over the additive model was 6.5% (*P* = 9 × 10^-4^) in scenario 1 but this advantage was not significant (*P*=0.84) in scenario 2. In scenario 3, where overdominance was absent, the proportion of *V*_*D*_ was only 5% and the realized heterosis was 8.4%. In this situation, responses to all three GS models were not significantly different (*P* > 0.37). In scenario 4, where there was no dominance, the dominance model still had a response as high as the additive model, which was 6.2% greater than the response for BSAM in generation 20 (*P* = 4 × 10^-8^).

**Table 1:** Cumulative response to genomic selection at generation 20 by the BSAM and dominance models compared to the additive model

	**One chromosome**		**Ten chromosomes**	
**Scenario**	**Dominance model**	**BSAM**	**Dominance model**	**BSAM**
1	22.4%*	6.5%*	10.1%*	1.7%
2	8.6%*	0.3%	2.1%	-3.1%
3	0.2%	-1.7%	1.0%	-4.4%
4	-0.1%	-5.9%*	-0.7%	-6.6%*

^*^Significant difference at the 0.01 level.

Cumulative responses were measured as the mean advantage of the additive model based on 1600 replicates of the simulations; the four scenarios are: (1) large overdominance, (2) realistic overdominance, (3) realistic incomplete dominance and (4) additive; in all scenarios, there was either one chromosome, 100 QTL and 1000 SNPs or ten chromosomes, 1000 QTL and 10 000 SNPs.

**Figure 3:**
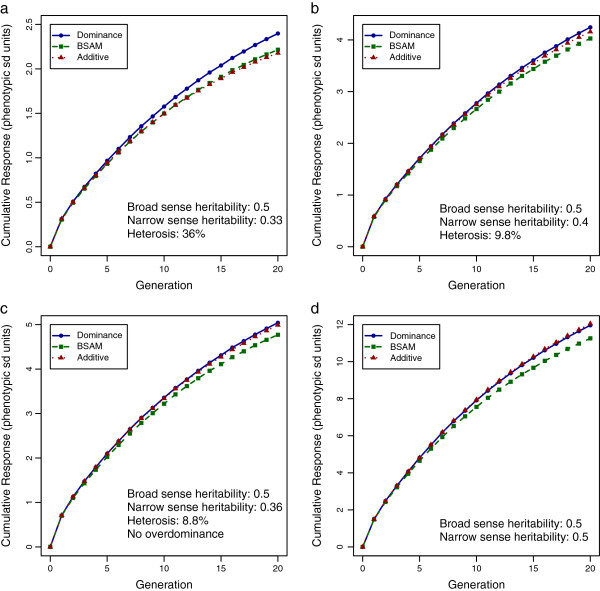
**Cumulative response to genomic selection with ten chromosomes.** Cumulative response to GS was computed using the dominance model, BSAM and the additive model in the four scenarios, when there were ten chromosomes, 1000 QTL and 10 000 SNPs; the plotted cumulative responses are means from 1600 replicates, standardized by the phenotypic standard deviation of crossbreds in generation 0: (**a**) results from scenario 1, where *V*_*D*_ was large with overdominance, (**b**) results from scenario 2, where *V*_*D*_ was realistic with overdominance, (**c**) results from scenario 3, where *V*_*D*_ was realistic without overdominance and (**d**) results from scenario 4, where dominance was absent.

Figure 3 shows the results with ten chromosomes, 1000 QTL and 10 000 SNPs in the genome. In scenario 1, where the dominance variance and the heterosis were set to be large, the dominance model had a clear advantage over BSAM and the additive model (*P* < 10^-10^). In the other scenarios, the dominance model had either the highest response or a response equal to that of the additive model, whereas BSAM was inferior to the additive model in most situations. Even in scenario 1, with large dominance variance, BSAM had merely a small non-significant advantage (*P* = 0.16) over the additive model. It can be seen from Table 1 that with more chromosomes and loci, the advantage of the dominance model over the additive model by generation 20 decreased, except in scenario 3, which had only incomplete dominance. The performance of BSAM was negatively affected by the increase in the number of loci in all scenarios. Although the differences between models became less significant in the simulations with ten chromosomes, the ranking of the models was consistent with those from the simulations with one chromosome.

### Changes in breed averages and heterosis in response to selection

From the definition of heterosis, cumulative response to selection in crossbred performance (CR) can be written as

**Figure 4:**
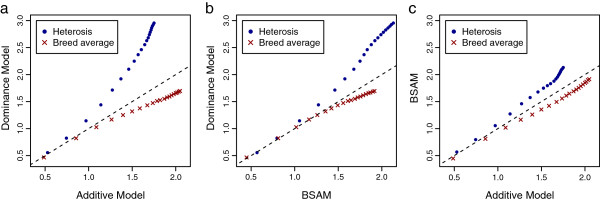
**Cumulative response for breed average and heterosis in crossbreds.** Cumulative response for breed average (red crosses) and heterosis in crossbreds (blue dots) was computed from scenario 1, with large *V*_*D*_ and allowing overdominance, when there was one chromosome, 100 QTL and 1000 SNPs; the plotted cumulative responses are means from 1600 replicates, standardized by the phenotypic standard deviation of crossbreds in generation 0: (**a**) cumulative response using the dominance model (y-axis) plotted against response using the additive model (x-axis), (**b**) cumulative response using the dominance model against response using BSAM and (**c**) cumulative response using BSAM against the additive model; the broken line is y=x.

CR=BA+H,

where BA denotes the breed average and H the heterosis manifested in the crossbreds. Thus, the observed advantage of the dominance model in some scenarios may be due to greater response in BA or in H, or in both. The relative contributions of BA and H to CR were investigated by partitioning CR into the response in BA and H for each of the 20 generations, and the differences between models were shown by plotting the results of selection on GEBV from one model against those of another model (Figure 4). Only results from scenario 1 with a single chromosome were used for illustration. In Figure 4, the advantage of a model in heterosis was always accompanied by some cost to purebred improvement. The dominance model had a lower response in BA, especially compared to the additive model. However, this lower response was more than compensated by increased heterosis, which resulted in a greater overall CR. By generation 20, the dominance model had a BA that was 0.35 phenotypic standard deviation (sd) lower than that of the additive model. This loss, however, was made up by an advantage of 1.2 phenotypic sd in heterosis, summing up to a total benefit of 0.95 phenotypic sd in CR for the dominance over the additive model (Figure 2a). In contrast, the advantage of BSAM over the additive model in heterosis was almost cancelled out by a comparable loss in response in BA (Figure 4c). This explains why BSAM had a limited advantage in CR over the additive model.

### Response to selection in heterozygosity

**Figure 5:**
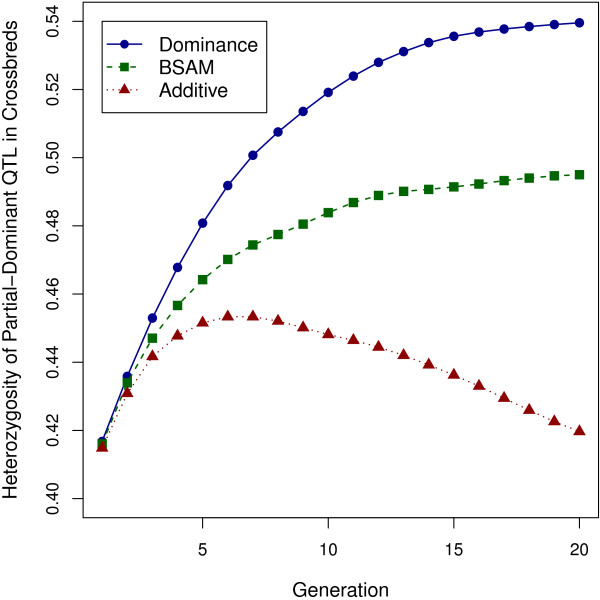
**Changes in heterozygosity for over-dominant QTL in crossbreds over generations.** The plotted values are changes in heterozygosity for over-dominant QTL in crossbreds over generations of selection, under the dominance model, BSAM and the additive model, in scenario 1, with one chromosome and large dominance, averaged over 1600 replicates.

When overdominance is present, crossbred performance is maximized when alternate alleles are fixed in the two pure breeds. Then, all crossbreds will be heterozygous for the over-dominant QTL. Genomic selection had a dramatic effect on heterozygosity of over-dominant QTL in crossbreds (Figure 5). In scenario 1, the dominance and BSAM models steadily increased heterozygosity over the 20 generations. However, the rate of increase was smaller for BSAM, for which heterozygosity stabilized to a lower level than for the dominance model after about 12 generations of selection because there was no retraining of the prediction model. With the additive model, heterozygosity increased up to generation 6 and then dropped in subsequent generations.

**Figure 6:**
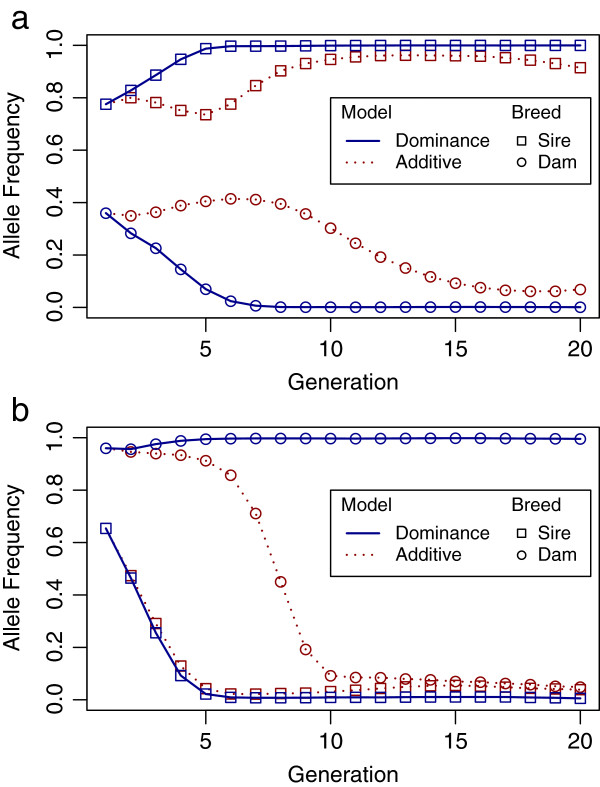
**Changes in allele frequencies of two over-dominant QTL in the parental breeds over generations.** The plotted values are changes in allele frequencies of two over-dominant QTL with major dominance effects in the sire and dam breeds over generations of selection, under the additive model and the dominance model, in scenario 1, with one chromosome and large dominance; results are means from 100 replicates in a given random simulation: (**a**) shows alternate alleles approaching fixation in the sire and dam breeds more rapidly with the dominance than the additive model and (**b**) shows the same allele approaching fixation in both parental breeds with the additive model, in contrast to the dominance model.

Figure 6 shows the response to selection in allele frequencies of two over-dominant QTL in the two parental breeds, where the plotted values are the means of 100 replicates of the selection process in a given random simulation. The advantage of the dominance model over the additive model had two components. First, the rate of fixation of alternate alleles was faster with the dominance model. Second, the same allele was more often fixed in both parental breeds with the additive model, which is undesirable for over-dominant QTL. The greater efficiency of the dominance model in fixing alternate alleles in the two breeds at over-dominant QTL explains the greater heterosis in Figure 4a.

## Discussion

Including dominance in addition to additive effects in the model was advantageous for response to selection when dominant gene action was present. Even when all gene action was purely additive, using the dominance model did not show a negative effect. However, an advantage of BSAM was observed only when dominance variance and heterosis were large, which confirms our hypothesis that the superiority of BSAM over the additive model may be primarily due to dominance effects, rather than differences in LD between the parental breeds.

### Comparison between the dominance model and the additive model

It has been shown [14] that for a two-way cross and ignoring selection, the allele substitution effects for QTL or markers in one parental breed depend on the allele frequencies in the other parental breed. Thus, in the computation of substitution effects, failure to use the appropriate allele frequencies may result in a loss of response to selection. Dekkers [23] showed that, with the availability of only purebred data, selection within a breed using QTL allele substitution effects that are based on the allele frequencies from the opposite breed gave substantially greater response to selection than using allele frequencies from the same breed.

In the additive model, a single substitution effect was estimated for each SNP, assuming it is the same for both parental breeds. Selection on GEBV derived using such allele substitution effects is expected to fix the favorable allele in both breeds (Figure 6a). Exceptions to this could be genetic drift or the marker and QTL being in LD with opposite phases in the two parental breeds (Figure 6b). When the two breeds have opposite LD phases, and a common nonzero substitution effect is estimated for a SNP in the additive model, the allele frequencies of associated QTL will move in opposite directions in the two breeds.

In the dominance model, breed-specific allele substitution effects were computed using estimated dominance and additive effects, together with the appropriate allele frequencies from the other parental breed. When overdominance is present, the allele substitution effect *α* = *a* + (1 - 2*p*)*d* may have opposite signs in the parental breeds, depending on allele frequencies *p* in the two breeds [12]. In this case, the two parental breeds are expected to be fixed for alternate alleles of over-dominant QTL, which increases the frequency of favorable heterozygotes in crossbred progeny. Note that under the additive model, fixation of the favorable allele in both breeds would result in lower heterozygosity in the crossbreds. This explains why the dominance model resulted in substantially greater heterosis than the additive model (Figure 4a). Recall that the purebred gain was lower with the dominance model than with the additive model because the unfavorable allele was moved towards fixation in one parental breed at some loci. However, the crossbred gain was more than compensated by the larger amount of heterosis in the crossbreds. The dominance model was mostly favored in crossbred gain in scenario 1, where the dominance variance was large (Figure 2a), because in this scenario the difference in allele substitution effects between breeds was large.

When dominance is incomplete, the breed-specific substitution effects for a locus will have the same sign in both breeds, as can been seen from (13). In the long term, and ignoring drift, the favorable allele will be fixed in both breeds with any GS model. Thus, differences in response to selection in scenario 3 were not significant between GS models (Figure 2c). However, Kinghorn et al. [11] observed that with many QTL, even incomplete dominance had a significant influence on the divergence of allele frequencies in the parental breeds because the effect of drift is not negligible with a large number of loci. This phenomenon was also confirmed in our study, in which the advantage of the dominance model over the additive model in cumulative response to selection increased from 0.2% to 1.0% in the presence of only incomplete dominance (Table 1, scenario 3), when the number of loci increased ten-fold. Although the signs of the substitution effects are the same in the parental breeds with incomplete dominance, their magnitude can be very different depending on the allele frequencies. In the additive model, where a common substitution effect is used, loci with negligible substitution effects will be under higher selection pressure than in the dominance model, where breed-specific substitution effects are used. When the number of loci is large, relative to the additive model, the dominance model will put little or no selection pressure on loci with small or negligible substitution effects, resulting in greater genetic progress. Thus, the ability to exploit dominance for loci without overdominance may still be very important.

The advantage of the dominance model over the additive model is attributable to the use of breed-specific allele substitution effects to calculate GEBV of purebreds for crossbred performance with the dominance model. Kinghorn et al. [10,11] observed an advantage of using breed-specific allele substitution effects for crossbreeding (reciprocal recurrent genomic selection, RRGS) in an ideal situation, where additive and dominance effects at the QTL were known without error. In the presence of overdominance, RRGS had greater cumulative response to selection than the additive model, regardless of whether recalculation of substitution effects of QTL alleles was performed only in the first generation, every five generations or every generation [10]. In the absence of overdominance, the advantage of RRGS over the additive model was still observed with retraining each generation, especially for many QTL [11].

### Comparison between BSAM and the additive model

In BSAM, for the first generation after training, the estimated breed-specific allele substitution effects of SNPs account for differences in LD and allele frequencies between breeds. However, with repeated selection over generations, the breed-specific allele substitution effects must be re-estimated in BSAM to accommodate changes in allele frequency and LD. Ibáne~z-Escriche et al. [9] showed that, when using crossbred data to predict GEBV of purebred descendants, the additive model had a greater accuracy of prediction than BSAM if the breeds were related and the training population size was small relative to the number of markers. However, pure additive inheritance was assumed in their study. Under dominant gene action, we expect BSAM to have an additional advantage over the additive model because, as can be seen from (13), apart from different LD, breed-specific allele substitution effects will be different when allele frequencies differ between breeds. However, in this study, BSAM had a lower response to selection than the additive model, except when the dominance effects were large and the number of SNPs was not greater than the number of observations. When the number of SNPs was ten times greater than the number of observations, the advantage of BSAM over the additive model was not significant.

One reason for the inferior performance of BSAM in most scenarios is model complexity. If a SNP is segregating in both parental breeds, then the SNP will have a nonzero substitution effect in the crossbreds for both breeds. Thus, when most SNPs are segregating in both breeds, we expect BSAM to have about twice the number of nonzero SNP effects in the model as the additive model. As shown in Table 2, the posterior mean of the number of effects in the additive model was proportional to the magnitude of the dominance variance relative to the additive variance, but this relationship was not observed in BSAM, for which the number of nonzero effects hardly changed between scenarios. In scenario 1, where the dominance variance was about half of the additive variance, many markers were needed in the additive model to jointly pick up the QTL effects. In this case, BSAM was superior to the additive model since only a few additional effects were fitted in the model. In scenario 4, where the size of additive variance was maximum, the number of effects in the additive model was reduced to about the same as the number of QTL, while the number of effects in BSAM was still higher than twice the number of QTL because the allele substitution effects were expected to be nonzero for both breeds. When the number of chromosomes and loci increased ten-fold, the performance of BSAM compared with the additive model became even worse due to the increased model complexity (Table 1).

**Table 2:** Posterior means of the number of nonzero SNP effects fitted in the model

		**BSAM**			**Dominance model**		
**Scenario**	**Additive model**	***α***^***S***^	***α***^***D***^	**Total**	***a***	**d**	**Total**
1	183.1	73.2	131.0	204.2	284.3	58.4	342.7
2	166.3	88.2	151.3	239.5	181.8	58.1	239.9
3	130.9	68.7	114.4	183.1	149.4	34.8	184.2
4	105.4	90.9	144.8	235.7	116.3	4.1	120.4

Numbers in the table are means from 16 random simulations, where there was one chromosome, 100 QTL and 1000 SNPs; *α*^*S*^ and *α*^*D*^ are the breed-specific allele substitution effects for the sire and dam breeds in BSAM; *a* and *d* are the additive and dominance SNP effects in the dominance model; numbers in column “Total” in BSAM and the dominance model are the sum of the number of nonzero SNP effects of different types in the corresponding model.

Another possible reason to explain the lower response of BSAM relative to the additive model was given by [9]. Consider a locus that is segregating in breed A but fixed in breed B. Because the additive model regresses phenotypes only on segregating alleles, the common substitution effect for this locus is actually the effect specific to breed A, just as in BSAM. However, BSAM includes an additional substitution effect for breed B, for which the allele is fixed. The substitution effect estimated from BSAM for the breed B allele will only add noise to the prediction of GEBV. Therefore, when several loci are nearly fixed in one of the parental breeds but segregating in the other, the additive model is expected to show an advantage over BSAM.

### Comparison between BSAM and the dominance model

The number of effects in BSAM equals the number of breeds times the number of SNPs. When only two breeds are involved in the cross, the dominance model is expected to have the same number of parameters as BSAM if the number of SNPs included in the model is fixed. However, with separate probability of inclusion parameters *Π*_*a*_ and *Π*_*d*_ for additive and dominance effects, the number of dominance effects that are fitted in the model only depends on the actual number of dominance effects for the trait. As dominance variance was lowered from large to null, the posterior mean of the number of nonzero dominance effects in the model decreased from 58.4 to 4.1 (Table 2). Thus, in contrast to BSAM, model complexity does not seem to be an issue for the dominance model. Even when dominance was absent, the response in the dominance model could not be distinguished from that of the additive model (Figure 2d) because only a few nonzero dominance effects were fitted in the model. In this regard, the dominance model is more robust than BSAM.

Even when additive and dominance effects are consistent between breeds, allele substitution effects will be breed-specific if allele frequencies differ between breeds. In such a case, the estimates of breed-specific allele substitution effects in the dominance model are expected to be more accurate than those in BSAM for the following four reasons:

First, the estimates of additive and dominance effects from the dominance model are combined with the observed allele frequencies in the opposite parental breed to calculate the breed-specific allele substitution effects. In BSAM, however, breed-specific allele substitution effects are estimated directly. Thus, the allele frequencies used in BSAM are based on the frequencies in the training population of the alleles inherited from the opposite parental breed. Note that the alleles inherited by the training population are a random sample of those from the parental population, and therefore their frequencies will deviate from those of the parental population. Thus, the use of observed allele frequencies from the parental population to compute breed-specific allele substitution effects favors the dominance model over BSAM.

Second, in the dominance model, as selection progresses and allele frequencies change due to selection, the observed allele frequencies in each generation are combined with the estimates of additive and dominance effects obtained in training to compute the current values of the breed-specific allele substitution effects. However, with the additive model and with BSAM, the allele substitution effects estimated in training are repeatedly used to compute GEBV of selection candidates, ignoring changes in allele frequencies. As a result, drops in accuracy of GEBV in generations of selection were greater for the additive model and BSAM than for the dominance model (results not shown). Thus, use of the dominance model is expected to require less frequent retraining than use of BSAM or the additive model. This is appealing for traits that are difficult or expensive to measure.

Third, under the assumption that additive and dominance effects of QTL are consistent across breeds, as explained below, the goodness of fit to training data is expected to be better for the dominance model than for BSAM. The expected phenotypic value for a given genotype *ij* at a QTL is,

(14) $$\begin{array}{ll} E(y|\text{ij})=G_{\text{ij}}=\left\{\begin{array}{ll} G_{00}, & \text{ij}=00\\ G_{00}+a+d, & \text{ij}=01\;\text{or}\;10\\ G_{00}+2a, & \text{ij}=11. \end{array}\right) & \; \end{array}$$

In the dominance model given by (3), these expected values are modeled exactly in terms of *a* and *d* as *μ* + *Xa* + *Wd*, where *X* = *W* = 0 for *ij* = 00, *X* = *W* = 1 for *ij* = (01 or 10), or *X* = 2 and *W* = 0 for *ij* = 11. In BSAM, these conditional expectations are modeled as

(15) $$\begin{array}{ll} G_{00} & =\mu +\alpha_{1}^{A}+\alpha_{1}^{B}+\epsilon_{00},\; \end{array}$$

(16) $$\begin{array}{lll} G_{01} & =\mu +\alpha_{1}^{A}+\alpha_{2}^{B}+\epsilon_{01},\;\\ G_{10} & =\mu +\alpha_{2}^{A}+\alpha_{1}^{B}+\epsilon_{10},\; & \;\\ G_{11} & =\mu +\alpha_{2}^{A}+\alpha_{2}^{B}+\epsilon_{11}.\; & \; \end{array}$$

Subtracting (16) from (15) gives

$$G_{01}-G_{00}=\alpha_{2}^{B}-\alpha_{1}^{B}+\epsilon_{01}-\epsilon_{00}.$$

As $\alpha^{B}=\alpha_{2}^{B}-\alpha_{1}^{B}$, based on (13),

$$\begin{array}{lll} G_{01}-G_{00} & =\alpha_{2}^{B}-\alpha_{1}^{B}+\epsilon_{01}-\epsilon_{00}\; & \;\\ & =a+d-2p^{A}d+\epsilon_{01}-\epsilon_{00}.\; & \; \end{array}$$

Comparing this to (14), where *G*_01_ - *G*_00_ = *a* + *d*, implies

$$\epsilon_{01}-\epsilon_{00}=2p^{A}d.$$

Thus, deviates *ϵ*_00_ and *ϵ*_01_ cannot both be zero. Similarly, it can be shown that *ϵ*_10_ - *ϵ*_00_ = 2*p*^*B*^*d* and *ϵ*_11_ - *ϵ*_00_ = 2(*p*^*A*^ + *p*^*B*^ - 1)*d*. The nonzero differences between the deviates imply that at least some deviates are not equal to zero. More simply, $\mu +\alpha_{1}^{A}+\alpha_{2}^{B}$ in the BSAM for *G*_01_ may not be equal to $\mu +\alpha_{2}^{A}+\alpha_{1}^{B}$ for *G*_10_, although *G*_01_ is equal to *G*_10_. In other words, the genotypic value is not exactly modeled in BSAM.

Forth, implementation of BSAM requires knowing the breed origins of SNP alleles but this is not required for the dominance model. In this study, breed origins of SNP alleles were assumed to be known without error but this may not hold for real data, which would introduce errors to the estimation of breed-specific SNP effects in BSAM.

However, the advantages of the dominance model may not hold if additive and dominance effects of SNPs are not consistent between breeds. More precisely, although *a* and *d* at the QTL may be consistent between breeds, the SNP effects may differ between breeds due to differences in LD. When these differences of SNP effects between breeds are large, the dominance model may become inferior to BSAM, for which breed-specific allele substitution effects account for differences in LD in addition to differences in allele frequencies. The magnitude and phase of LD, especially long-range LD, has been found to be inconsistent between breeds in livestock [24-26]. However, whether those differences in LD are large enough to enable BSAM to outperform the dominance model needs further investigation with real data.

The benefit of using one model over another depended on the number and size of QTL and the density of SNPs relative to the size of the training population. When the genome was extended from one to ten chromosomes, there was a ten-fold increase in the number of SNPs but the SNP density remained the same. In addition, with the values of variance components held constant, each QTL explained a smaller proportion of the total genetic variance for the larger genome, which made it more difficult to capture the QTL effects through SNPs. This explains the observed reduction of differences in response between models. This suggests that the preference of the dominance model is expected to hold for a larger genome but the amount of benefit will decrease when the genetic architecture is more polygenic. Furthermore, a larger training population will be needed.

In this study, SNP effects were estimated only once and applied successively over 20 generations of selection. This is rarely done in practice and retraining is usually carried out after each generation of selection. With retraining, the advantage of the dominance model is expected to be much smaller or may even be ignored because in that case, the additive and BSAM models are also expected to give estimated allele substitution effects using allele frequencies from the current or recent generations. However, if the training population consists of individuals from multiple generations, the estimates of substitution effects in the additive model or BSAM will depend on allele frequencies across multiple generations, which may not be appropriate to predict crossbred performance for the purebred candidates.

Although the dominance model was studied here when purebreds were selected for crossbred performance, the advantages of this model over the additive model also apply to selection for purebred performance. Furthermore, the estimates of *a* and *d* from the dominance model can be used to predict GEBV in any population with SNP genotypes, provided the LD in the training and candidate populations are about the same. It has been found that the accuracy of GEBV in Holstein-Friesian cattle with training in Jerseys and vice versa was as low as -0.1 to 0.3 over traits [27], and the correlations of SNP allele frequencies between Holsteins, Jerseys, and Brown Swiss cattle were as low as 0.65 to 0.67 [28]. Thus, differences in allele frequencies between breeds, along with dominant gene action, can be attributed to the low accuracy of prediction across breeds in dairy cattle. In this situation, the dominance model is expected to give better results, especially with a high marker density, since then LD would be more consistent between breeds.

Besides dominance, other types of non-additive effects may also contribute to heterosis, such as epistasis, imprinting, etc. The dominance model can be further extended to account for imprinting if the heterozygotes are phased. Epistatic interactions between loci are partially included in BSAM as a component of allele substitution effects but will be misspecified in the dominance model, which will impair accuracy of selection. Thus, BSAM may be more robust in the presence of epistasis.

## Conclusions

When dominance, particularly overdominance, is the key driver of heterosis, using a dominance model for GS is expected to result in greater cumulative response to selection of purebred animals for crossbred performance than either BSAM or the additive model. The extent of this additional response to selection depends on the size of dominance effects at the QTL and the power of detecting the dominance effects through SNP genotypes. Also, when there are many loci, it is important to exploit dominance, even in the absence of overdominance. When BayesC *Π* is used, the dominance model is robust because, even in the absence of dominance, it does not give a lower response than the additive model. BSAM is favored over the additive model only when dominance effects are large enough to overwhelm its shortcomings. Furthermore, implementation of BSAM requires that the breed origins of SNP alleles are known. Our results suggest that in the presence of dominant gene action, relative to BSAM and the additive model, GS with the dominance model is superior to maximize crossbred performance through purebred selection, especially when no retraining is carried out at each generation.

## Appendix A

### Scaling procedure for QTL additive and dominance effects

Let *V*_*A*_ and *V*_*D*_ denote the observed additive and dominance genetic variance of the trait. Assuming no genotype by genotype interactions among QTL that define the trait, the genetic variance components can be written as the sum of the variance explained by each QTL [12]:

(17) $$\begin{array}{ll} V_{A} & =\underset{j}{\sum }2p_{j}q_{j}\alpha_{j}^{2},\; \end{array}$$

(18) $$\begin{array}{ll} V_{D} & =\underset{j}{\sum }{\left(2p_{j}q_{j}d_{j}\right)}^{2},\; \end{array}$$

where *p*_*j*_ = 1 - *q*_*j*_ is the observed allele frequency for QTL *j*, *d*_*j*_ is the QTL dominance effect, and *α*_*j*_ is the QTL allele substitution effect defined as

$$\alpha_{j}=a_{j}+(q_{j}-p_{j})d_{j},$$

where *a*_*j*_ is the QTL additive effect.

#### For scenarios allowing overdominance

Let $V_{A}^{*}$ and $V_{D}^{*}$ denote the corresponding desired genetic variance components and $a_{j}^{*},d_{j}^{*},\alpha_{j}^{*}$ the corresponding scaled QTL effects. Let

(19) $$s=\frac{V_{D}^{*}}{V_{D}}.$$

From (18) and (19), we have

$$\underset{j}{\sum }{\left(2p_{j}q_{j}d_{j}^{*}\right)}^{2}=s\underset{j}{\sum }{\left(2p_{j}q_{j}d_{j}\right)}^{2}.$$

Thus,

$$d_{j}^{*}=\sqrt{s}d_{j}.$$

Similar to $\sqrt{s}$ the scalar for dominance effects, we have a scalar *t* for additive effects such that

(20) $$a_{j}^{*}=ta_{j},$$

and

(21) $$\begin{array}{lll} V_{A}^{*} & =\underset{j}{\sum }2p_{j}q_{j}{\left(\alpha_{j}^{*}\right)}^{2}\; & \;\\ & =\underset{j}{\sum }2p_{j}q_{j}{\left(a_{j}^{*}+(q_{j}-p_{j})d_{j}^{*}\right)}^{2}.\; \end{array}$$

Substituting (20) in (21) and rearranging it, we have

$$\begin{array}{lll} & t^{2}\underset{j}{\sum }2p_{j}q_{j}a_{j}^{2}+t\underset{j}{\sum }(q_{j}-p_{j})a_{j}d_{j}+\sum {\left(q_{j}-p_{j}\right)}^{2}d^{*2}\; & \;\\ & -V_{A}^{*}=0.\; & \; \end{array}$$

This can be seen as a quadratic equation with variable *t* unknown. Thus, the scalar *t* for the additive effects can be obtained by solving this equation.

#### For scenarios without overdominance

Since *d* is already smaller than *a* for each QTL, to obtain the desirable additive genetic variance $V_{A}^{*}$, we have a scalar *c* where

$$c=\frac{V_{A}^{*}}{V_{A}}.$$

Based on (17), the new substitution effect $\alpha_{j}^{*}$ for QTL *j* is simply

$$\alpha_{j}^{*}=\sqrt{c}\;\alpha_{j}.$$

Thus, the scaled additive and dominance effects are respectively, $a_{j}^{*}=\sqrt{c}a_{j}$ and $d_{j}^{*}=\sqrt{c}d_{j}$. With this approach, the additive genetic variance is the one desired but the dominance genetic variance is not under control. However, as desired, there would be no overdominance affecting the trait.

## Appendix B

### Hypothesis test for the GS model effect

The objective was to test if any difference in cumulative response to GS observed at generation 20 of selection among the additive model, BSAM, and the dominance model is statistically significant. As described, data were collected from 16 simulations each with 100 replicates resulting in a total of 1600 observations. The following mixed linear model was used to fit the data:

$$y_{\text{ij}}=\mu +m_{i}+b_{j}+e_{\text{ij}},$$

where *y*_*ij*_ is the response from GS model *i* in simulation *j*, *m*_*i*_ is the fixed effect for GS model *i* = {1, 2, 3}, $b_{j}∼N(0,\sigma_{b}^{2})$ is the random blocking effect for simulation set *j* = {1, …, 16}, and $e_{\text{ij}}∼N(0,\sigma_{e}^{2})$ is the residual. A set of t-tests was used for testing the null hypothesis that 1) *m*_1_ = *m*_2_, 2) *m*_1_ = *m*_3_, or 3) *m*_2_ = *m*_3_.

## Appendix C

### Full conditionals for some key model parameters

Let *β*_*j*_ denote either *a*_*j*_ or *d*_*j*_ in the dominance model. The mixture prior for *β*_*j*_ allows it to be included or not in the model. Let *δ*_*j*,*β*_ denote a model inclusion indicator variable defined as

$$\delta_{j,\beta }=\left\{\begin{array}{ll} 1 & \text{then}\;\beta_{j}∼\text{Normal}\\ 0 & \text{then}\;\beta_{j}=0 \end{array}\right)$$

with a prior probability 1 - *Π*_*β*_ that *δ*_*j*,*β*_ = 1. It should be clear that the model allows *δ*_*j*,*a*_ ≠ *δ*_*j*,*d*_. The indicator *δ*_*j*,*β*_ and the effect *β*_*j*_ can be sampled jointly by first sampling *δ*_*j*,*β*_ from its marginal distribution and then sampling *β*_*j*_ conditional on *δ*_*j*,*β*_. The posterior “marginal” probability that *δ*_*j*,*β*_ = 1 respecting to *β*_*j*_ is calculated by

(22) $$\begin{array}{lll} \text{Pr}(\delta_{j,\beta } & =1|y,\theta_{\text{else}})\; & \;\\ & =\frac{f(y|\delta_{j,\beta }=1,\theta_{\text{else}})\text{Pr}(\delta_{j,\beta }=1)}{\underset{\delta_{j,\beta }}{\sum }f(y|\delta_{j,\beta },\theta_{\text{else}})\text{Pr}(\delta_{j,\beta })},\; \end{array}$$

where ***θ***_*else*_ denotes other parameters besides *δ*_*j*,*β*_ and *β*_*j*_. The “likelihood” in (22) can be obtained by integrating *β*_*j*_ out from the sampling distribution as described below.

Let

$$\begin{array}{ll} v=y-1\mu -\mathrm{Xa}-\underset{j^{\prime }\neq j}{\sum }w_{j^{\prime }}d_{j^{\prime }}=w_{j}d_{j}+e. & \; \end{array}$$

Then,

(23) $$\begin{array}{lll} f & (y|\delta_{j,d}=1,\theta_{\text{else}})\; & \;\\ & =\int f(y|\delta_{j,d}=1,d_{j},\theta_{\text{else}})f(d_{j})\;dd_{j}\; & \;\\ & ={\left(2\Pi \right)}^{-\frac{n}{2}}{\left(\sigma_{e}^{2}\right)}^{-\frac{n}{2}}{\left(\sigma_{d}^{2}\right)}^{-\frac{1}{2}}{\left(\frac{C_{j,d}}{\sigma_{e}^{2}}\right)}^{-\frac{1}{2}}\; \end{array}$$

(24) $$\begin{array}{ll} & \;\exp\{-\frac{v^{\prime }v+\lambda \mu_{d}^{2}-C_{j,d}{\left({\hat{d}}_{j}+\lambda C_{j,d}^{-1}\mu_{d}\right)}^{2}}{2\overset{2}{\underset{e}{\sigma }}}\},\; \end{array}$$

where $C_{j,d}=w_{j}^{\prime }w_{j}+\frac{\sigma_{e}^{2}}{\sigma_{d}^{2}}$ and ${\hat{d}}_{j}=\frac{w_{j}v}{C_{j,d}}$ are, respectively, the coefficient of the mixed-model equation and the BLUP estimate for *d*_*j*_. Similarly, let

$$\begin{array}{lll} \;u & =y-1\mu -\mathrm{Wd}-\underset{j^{\prime }\neq j}{\sum }X_{j^{\prime }}a_{j^{\prime }}\; & \;\\ & =X_{j}a_{j}+e.\; & \; \end{array}$$

We have

(25) $$\begin{array}{lll} f(y|\delta_{j,a}=1,\theta_{\text{else}})= & {\left(2\Pi \right)}^{-\frac{n}{2}}{\left(\sigma_{e}^{2}\right)}^{-\frac{n}{2}}{\left(\sigma_{a}^{2}\right)}^{-\frac{1}{2}}{\left(\frac{C_{j,a}}{\sigma_{e}^{2}}\right)}^{-\frac{1}{2}}\; & \;\\ & \exp\{-\frac{u^{\prime }u-C_{j,a}{\left({â}_{j}\right)}^{2}}{2\sigma_{e}^{2}}\},\; \end{array}$$

where $C_{j,a}=X_{j}^{\prime }X_{j}+\frac{\sigma_{e}^{2}}{\sigma_{a}^{2}}$ and ${â}_{j}=\frac{X_{j}u}{C_{j,a}}$. For *δ*_*j*,*β*_ = 0, the “likelihood” is simply

(26) $$\begin{array}{ll} f(y|\delta_{j,\beta }=0,\theta_{\text{else}})={\left(2\Pi \right)}^{-\frac{n}{2}}{\left(\sigma_{e}^{2}\right)}^{-\frac{n}{2}}\exp\{-\frac{w^{\prime }w}{2\sigma_{e}^{2}}\}, & \; \end{array}$$

where ***w*** is either ***v*** or ***u*** corresponding to *δ*_*j*,*d*_ or *δ*_*j*,*a*_. Substituting (24-26) in (22) gives the marginal distribution of *δ*_*j*,*β*_ respecting to *β*_*j*_.

Given that *δ*_*j*,*d*_ = 1, *d*_*j*_ has a normal prior centered at *μ*_*d*_, otherwise it is zero. Let ***d***_-*j*_ denote the other additive effects besides that for SNP *j*, then the full conditional for *d*_*j*_ is

$$\begin{array}{lll} f(d_{j} & |y,\mu ,d_{-j},a,\mu_{d},\sigma_{d}^{2},\sigma_{e}^{2})\; & \;\\ & \propto f(y|\mu ,d_{-j},a,\sigma_{e}^{2})f(d_{j}|\mu_{d},\sigma_{d}^{2})\; & \;\\ & \propto \exp\left\{-\frac{C_{j,d}{\left\{d_{j}-\left({\hat{d}}_{j}+\frac{\lambda }{C_{j,d}}\mu_{d}\right)\right\}}^{2}}{2\sigma_{e}^{2}}\right\},\; & \; \end{array}$$

which is a normal $N({\hat{d}}_{j}+\frac{\lambda }{C_{j,d}}\mu_{d},\frac{\sigma_{e}^{2}}{C_{j,d}})$ where $\lambda =\frac{\sigma_{e}^{2}}{\sigma_{d}^{2}}$.

The variance variable for the dominance effect depends on the data only through the dominance effects:

$$\begin{array}{ll} f(\sigma_{d}^{2}|y,\mu ,a,d,\sigma_{e}^{2},S_{d})\propto f(d|\sigma_{d}^{2})f(\sigma_{d}^{2}|S_{d}). & \; \end{array}$$

Thus, due to the conjugacy, the full conditional for $\sigma_{d}^{2}$ is also a scaled inverse Chi-square $∼{\tilde{S}}_{d}\chi_{{\tilde{\nu }}_{d}}^{-2}$ where ${\tilde{\nu }}_{d}=k+\nu_{d}$ and ${\tilde{S}}_{d}=\frac{{\left(d-1\mu_{d}\right)}^{\prime }(d-1\mu_{d})+\nu_{d}S_{d}}{{\tilde{\nu }}_{d}}$ given the sampled value of *μ*_*d*_.

As shown, the prior of *μ*_*d*_ depends on the value of $\sigma_{d}^{2}$, therefore the full conditional for *μ*_*d*_ is

$$\begin{array}{lll} f(\mu_{d} & |y,\mu ,a,d,\sigma_{d}^{2},\sigma_{e}^{2},\eta ,\phi )\; & \;\\ & \propto f(d|\mu_{d},\sigma_{d}^{2})f(\mu_{d}|\eta ,\phi ,\sigma_{d}^{2})\; & \;\\ & \propto \exp\{-\frac{{\left(\mu_{d}-\frac{1^{\prime }d+\phi \eta }{k+\phi }\right)}^{2}}{2\overset{2}{\underset{d}{\sigma }}/(k+\phi )}\},\; & \; \end{array}$$

which is a normal $∼N\left(\frac{1^{\prime }d+\phi \eta }{k+\phi },\frac{\sigma_{d}^{2}}{k+\phi }\right)$.

The full conditionals for the other parameters including additive effect for SNP *j* and its variance variable are as illustrated in [6].

## Competing interests

The authors declare that they have no competing interests.

## Authors’ contributions

JZ and RLF designed the study and developed the algorithm for the dominance model. JZ and AT wrote the simulation programs under RLF’s guidance. JZ carried out the simulation studies, performed the statistical analysis, and drafted the manuscript. JCMD and DJG advised on the design of the study, the development of the algorithm, and the analysis of the simulations. RLF, JCMD and DJG revised the manuscript. All authors read and approved the final manuscript.
